# *Mycobacterium shimoidei* causing granulomatous inflammation in a captive bird species, the white-cheeked turaco

**DOI:** 10.1177/10406387261471130

**Published:** 2026-07-30

**Authors:** Sara Pagliarani, Liz Orkhulik, Kate Hartzler, Chiara Palmieri, Olga Andrievskaia, Mirjana Savic, Mauricio Seguel

**Affiliations:** Department of Pathobiology, Ontario Veterinary College, University of Guelph, Guelph, Ontario, Canada; Toronto Zoo, Scarborough, Ontario, Canada; Toronto Zoo, Scarborough, Ontario, Canada; The University of Queensland, School of Veterinary Science, Gatton, Queensland, Australia; Ottawa Animal Health Laboratory, Canadian Food Inspection Agency, Ottawa, Ontario, Canada; Ottawa Animal Health Laboratory, Canadian Food Inspection Agency, Ottawa, Ontario, Canada; Department of Pathobiology, Ontario Veterinary College, University of Guelph, Guelph, Ontario, Canada

**Keywords:** avian mycobacteriosis, *Mycobacterium shimoidei*, non-tuberculous mycobacterium, white-cheeked turaco

## Abstract

Mycobacteriosis in avian species is generally caused by *Mycobacterium avium* subsp. *avium* and *M. genavense*, although sporadic reports also exist of other nontuberculous mycobacterium species in birds. Here, we describe disseminated mycobacteriosis caused by *M. shimoidei* (a rare and infrequently reported pathogen in humans and animals) in a bird species. A captive, adult white-cheeked turaco (*Tauraco leucotis*) was presented because of persistent dyspnea unresponsive to treatment. The animal was treated unsuccessfully with antimicrobials and later euthanized on welfare grounds. At autopsy, granulomas were identified in the lungs and spleen. Histologically, the coalescing granulomas with central necrosis contained numerous chains of slender, slightly curved, 2-µm acid-fast bacilli, suggesting mycobacterial infection. Bacterial isolation confirmed *Mycobacterium* spp., which was identified as *M. shimoidei* by partial sequencing of the 16S rRNA and *rpoB* genes. Our report expands the known host range of *M. shimoidei* and highlights the importance of molecular testing methods in identifying atypical nontuberculous mycobacteria (NTM) in zoologic collections. Our case also underscores the need for enhanced epidemiologic surveillance of NTM infections in avian species, given their potential implications for both animal and public health.

Avian mycobacteriosis, often referred to as avian tuberculosis, is a chronic granulomatous disease caused by a range of nontuberculous mycobacterial (**NTM**) species. Although *Mycobacterium avium* subsp. *avium* is the most common pathogen in poultry,^18^ other species, such as *M. genavense*, predominate in pet and captive birds,^8,13^ including psittacine species.^10^ Other NTMs, such as *M. fortuitum* and *M. intracellulare*, have been reported in birds housed in zoologic collections.^13^ In turacos, avian mycobacteriosis has been sporadically reported in captive birds in the United States and Europe,^1,2,15,17^ with *M. avium* complex identified in a minority of cases.

Diagnoses often rely solely on gross or histologic findings; antemortem diagnosis remains a major hurdle. Common screening tools—such as the intradermal tuberculin test, serologic assays, routine hematology, and radiography—lack the sensitivity and/or specificity needed for reliable individual diagnosis.^18^ Consequently, definitive diagnosis often depends on postmortem examination, which enables detection of characteristic granulomatous lesions and facilitates confirmation through acid-fast staining, culture, or PCR. Although lesions typically affect liver, spleen, lung, and intestine, atypical or localized infections of skin, bone, or ocular tissues also are described.^7,9,14^ Zoo surveillance data indicate that, although mycobacteriosis can recur within aviaries, it usually affects birds sporadically rather than as explosive outbreaks.^1^

*M. shimoidei* is a rare, slow-growing NTM first isolated from a human patient in Japan in 1975^20^ and recognized as a distinct species in 1982. Human infections are rare, usually involve pulmonary disease in immunocompromised individuals, and require molecular methods for confirmation.^5^ To date, *M. shimoidei* has been reported only once in a non-human host: a goldfish (*Carassius auratus*) housed in a freshwater aquarium in Pretoria, South Africa, which was presented with granulomatous intestinal lesions.^4^

White-cheeked turacos (*Tauraco leucotis*; family *Musophagidae*) are medium-sized, frugivorous birds native to the forests of Ethiopia, Eritrea, Sudan, and South Sudan.^2^ They are commonly kept in zoologic collections given their vibrant plumage and relatively calm behavior. These birds thrive in tree canopies and are adapted to arboreal living, with strong feet for gripping branches and a diet composed largely of fruit. In captivity, they can live for up to 30 y and are considered long-lived among zoo-housed members of the family *Musophagidae*.^2^ Although nutritional deficiencies or traumas are common health issues in captive populations, infectious diseases such as avian mycobacteriosis also pose a significant risk.^1^ The relatively long lifespan and low reproductive output of turacos increase the significance of individual losses in small zoologic collections. Thus, understanding the disease dynamics in these birds is critical for informed management and biosecurity protocols.

Here, we document a case of *M. shimoidei* in a bird, with detailed clinicopathologic and microbiologic findings.

An 18-y-old female white-cheeked turaco was presented to the Toronto Zoo Wildlife Health Centre (Scarborough, ON, Canada) for evaluation of persistent dyspnea refractory to supportive care and antimicrobial and antifungal therapy. Treatment included enrofloxacin (Baytril), terbinafine (compounded formulation; Chiron Compounding Pharmacy), and subsequently clotrimazole. Aspergillosis was considered the primary differential diagnosis based on clinical presentation and partial therapeutic response. The bird was housed in a mixed-species aviary with outdoor access and had recently been reintroduced to a bat colony. Another conspecific from the same aviary had undergone prolonged treatment for suspected aspergillosis and, despite initial improvement, died suddenly; postmortem examination revealed severe mycotic pneumonia, although fungal speciation was not performed. CBC revealed marked leukocytosis (71.1 × 10^9^/L; RI: 0.9–18.2 × 10^9^/L), mild heterophilia (10.7 × 10^9^/L; RI: 0.4–6.0 × 10^9^/L), and marked monocytosis (52.6 × 10^9^/L; RI: 0.02–1.9 × 10^9^/L).

Respiratory signs improved transiently; open-mouth breathing, hyporexia, and generalized weakness developed. Given the poor prognosis, euthanasia was elected. Postmortem radiographs revealed a large pulmonary mass. Autopsy revealed a 1–1.5-cm, well-demarcated, tan, firm nodule in the caudal right lung (**Fig. 1**). The cranial right lung, left lung, trachea, and syrinx were grossly unremarkable. Pinpoint tan nodules were present along the air sac margins. Other celomic organs, including the spleen, were within normal limits. Selected tissues were fixed in 10% neutral-buffered formalin and routinely processed for histology. Selected organs were also sampled and stored at −80°C. Histological sections were stained with H&E and the Ziehl–Neelsen acid-fast method.

**Figure 1.**
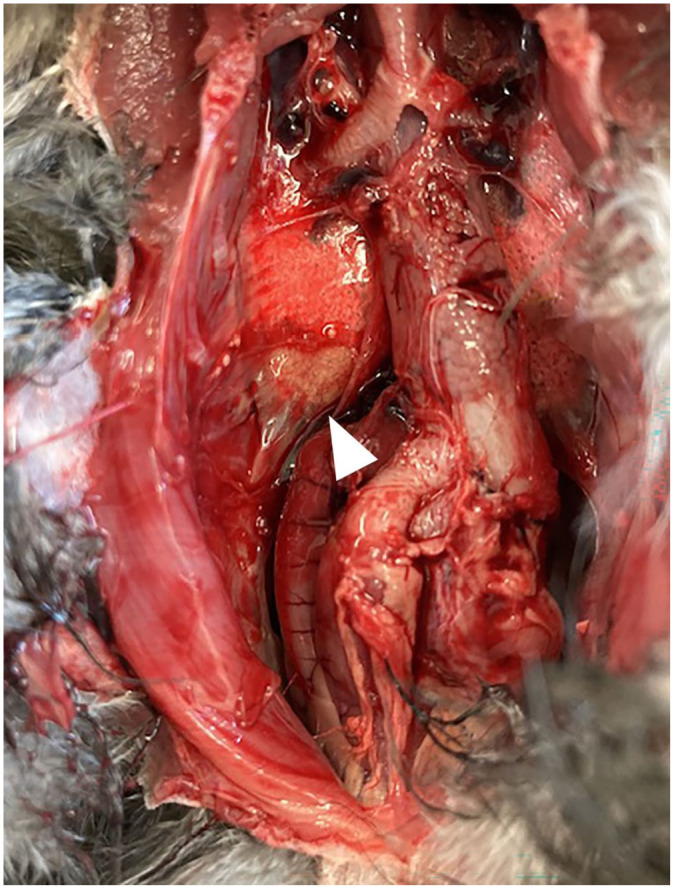
*Mycobacterium shimoidei* infection in a white-cheeked turaco. A single well-demarcated, 1–1.5-cm, tan nodule (arrowhead) in the caudal third of the right lung.

Microscopically, coalescing granulomas effaced 40% of the lung parenchyma (**Fig. 2**). These granulomas were composed of a central caseous necrosis with basophilic mineralized material surrounded by epithelioid macrophages, multinucleate giant cells, and a few peripheral lymphocytes. Numerous 2–3-µm acid-fast rods were present within the granuloma cores. Similar granulomas containing acid-fast rods were found in the spleen. No granulomatous lesions or acid-fast organisms were identified in the intestines, either grossly or histologically. Representative frozen sections of lung and spleen were submitted to the Ottawa Animal Health Laboratory, Canadian Food Inspection Agency (OAHL, CFIA; Ottawa, ON, Canada), where mycobacterial isolation and identification was performed according to standard procedures. Primary bacterial isolation was performed using several types of liquid and solid media. After subculture on modified Middlebrook medium supplemented with malachite green and on Lowenstein–Jensen medium, non‑chromogenic colonies became visible after 3 wk of incubation at 37°C, 42°C, and 45°C, with optimal growth observed at 37°C. Molecular identification, based on partial sequencing of the 16S rRNA and *rpoB* genes,^6^ revealed the highest similarity to *M. shimoidei*, with 99.1% identity across a 960-bp fragment of the 16S rRNA gene and 100% identity across a 715-bp fragment of the *rpoB* gene.

**Figure 2.**
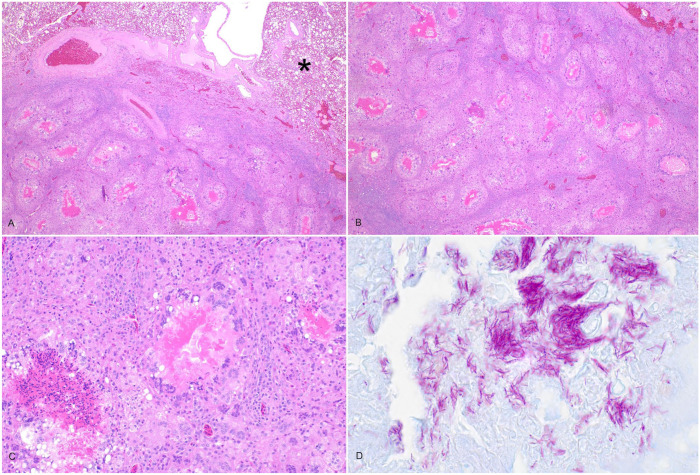
Microscopic lesions of *Mycobacterium shimoidei* infection in the lung of a white-cheeked turaco. **A, B.** Granulomas with a necrotic center efface lung architecture. The asterisk (*) indicates adjacent normal lung parenchyma. H&E. **C.** A thin rim of epithelioid macrophages with scattered lymphocytes surrounds an area of caseous necrosis. H&E. **D.** Chains of slender, acid-fast bacilli within areas of caseous necrosis in the lung. Ziehl–Neelsen stain.

Our case expands the known host range of *M. shimoidei.* In humans, *M. shimoidei* is an uncommon pulmonary pathogen, with <50 cases reported worldwide.^5^ Infections typically affect immunocompromised individuals and often are diagnosed only through molecular methods. Most affected human patients have preexisting lung diseases (such as chronic obstructive pulmonary disease or interstitial lung disease), previous *M. tuberculosis* or other NTM lung infection, and a history of heavy smoking.^5^ Unlike *M. avium* or *M. genavense*, this NTM species has not been reported in avian disease. To assess whether *M. shimoidei* infection had been reported previously in white-cheeked turacos, we searched Google Scholar, PubMed, Web of Science, and Scopus using combinations of the terms “Mycobacterium shimoidei”, “turaco”, “Tauraco leucotis”, “Musophagidae”, and “avian mycobacteriosis”. We retrieved no reports describing *M. shimoidei* infection in white-cheeked turacos, suggesting that this condition has not been reported previously in this species.

The pathogenesis of NTM infections in birds typically involves environmental acquisition through ingestion^16,19^ or, less frequently, by inhalation.^3^ The severe lung lesions and histologic findings in our case may support inhalation as the most likely route of entry, with subsequent hematogenous spread, supported by the presence of granulomas in the spleen. In humans, *M. shimoidei* infection has been associated with granulomatous lesions that may have cavitary features in the lungs.^5^ In contrast, in our avian case, granulomas were typically non-cavitary and instead formed solid, well-demarcated nodules. The bird’s exposure to outdoor aviaries, potential contact with soil and standing water, and possible contamination of feed or substrate all represent plausible sources of infection.

NTMs are notoriously resilient, persisting in the environment for months to years.^11^ NTMs are resistant to many disinfectants and thrive in biofilms and organic debris. Fecal shedding by infected birds has been described, creating further risk of environmental contamination and possible transmission within mixed aviaries.^15^ Although no evidence of bird-to-human transmission is reported for *M. shimoidei*, other mycobacteria are recognized zoonotic agents. Psittacine birds and other exotic species may harbor subclinical mycobacterial infections, posing diagnostic and management challenges.^10^ Diagnosis is often delayed because of the insidious onset and nonspecific signs, including lethargy, weight loss, or dyspnea. Molecular techniques have greatly improved our ability to identify rare NTMs.

Given the zoonotic potential of some NTM species and the increasingly close contact between humans and captive exotic birds, correct identification is important for One Health surveillance. Although *M. shimoidei* has not been linked to zoonotic transmission, the finding of this organism in a bird raises questions about environmental sources and cross-species exposure. Our case also raises the importance of routine autopsy and advanced laboratory testing in zoo mortalities. Without molecular confirmation, our case might have been misclassified as a generic *M. avium* subsp. *avium* infection. Unfortunately, the sequence of the isolate obtained was not deposited in GenBank. Prospective screening of avian populations for NTM carriage using PCR of droppings or cloacal swabs may help identify subclinical carriers and mitigate the risk of outbreaks. Various species of NTM have been detected in droppings or gastrointestinal tract samples from several bird species.^11,16^ Fecal shedding of NTM, including *M. shimoidei*, can serve as a key route of transmission, posing a risk of infection to other birds, animals, or humans through direct contact or indirect contamination of the environment, food, or water.

A 2020 report of *M. avium* subsp. *hominissuis* (**MAH**) infection in a red-crested turaco (*Tauraco erythrolophus*) highlights a parallel case involving a closely related host species.^1^ In that case, molecular techniques identified an INMV 51 strain of MAH, a mycobacterium commonly found in humans, pigs, and cattle; autopsy of the emaciated bird revealed granulomas within the body cavity and inner organs.^1^ Although the host species and mycobacterial isolate differed, both cases emphasize that turacos can harbor atypical NTMs not previously considered significant in avian pathology. Such findings underscore the critical need for thorough mycobacterial speciation in avian cases and for expanded surveillance to determine the prevalence of such infections across zoologic institutions.

Given that *M. shimoidei* has been isolated from environmental samples in aquatic systems, it is possible that water features within enclosures could serve as reservoirs. Regular monitoring of water sources, especially in outdoor aviaries, may reduce the risk of NTM exposure. Research into host range, environmental persistence, and potential zoonotic risks of *M. shimoidei* is needed. The immunologic status of the host likely plays a critical role in susceptibility to NTM infections.^10^ Chronic stress, environmental fluctuations, or nutritional deficiencies common in captivity may impair host defense mechanisms, predisposing birds to progressive mycobacterial disease. Although we identified no overt immunosuppressive condition in our case, the chronicity of clinical signs suggests a failure to contain infection at mucosal barriers. Our case had also been introduced recently to a bat colony within its enclosure, which may have been an additional stressor. Notably, an investigation of NTM mycobacteria in bat guano found a high prevalence of mycobacterial DNA and culture confirmed isolates, including *M. fortuitum*, *M. chelonae*, and members of the *M. avium*, *M. terrae*, *M. vaccae*, and *M. smegmatis* complexes, although none was categorized as a high-risk, obligatory pathogen. Bat guano could serve as an environmental reservoir for mycobacterial exposure.^12^ Unfortunately, fecal samples from the bat colony were not available for analysis in our case; their presence was only recognized after the case investigation.

Although the bird received supportive care, including supplemental oxygen and antibiotics targeting common respiratory pathogens, the antimicrobial therapy showed limited efficacy. Treatment of NTM infections in birds is difficult given the difficulty in choosing effective active compounds, antimicrobial resistance, and the need for prolonged therapy. In humans, antimicrobial susceptibility testing for isolated *M. shimoidei* has been performed on a case-by-case basis to guide treatment.^5^

Our case highlights the diagnostic challenges of avian mycobacteriosis and the value of thorough postmortem examination combined with molecular techniques to identify atypical pathogens. In zoologic settings, in which individual losses can have significant implications for small populations, recognizing and characterizing emerging infectious agents is essential for disease management and biosecurity. Continued surveillance, environmental monitoring, and targeted diagnostic efforts will be critical to improving our understanding of *M. shimoidei* and its potential role in avian health.
